# TRIPLE C reporting principles for case study evaluations of the role of context in complex interventions

**DOI:** 10.1186/s12874-023-01888-7

**Published:** 2023-05-13

**Authors:** Sara E. Shaw, Sara Paparini, Jamie Murdoch, Judith Green, Trisha Greenhalgh, Benjamin Hanckel, Hannah M. James, Mark Petticrew, Gary W. Wood, Chrysanthi Papoutsi

**Affiliations:** 1 Nuffield Department of Primary Care Health Sciences, University of Oxford, Radcliffe Observatory Quarter, Oxford, OX2 6GG UK; 2 Wolfson Institute of Population Health, Queen Mary University of London, London, UK; 3 School of Life Course and Population Sciences, King’s College London, London, UK; 4 Wellcome Centre for Cultures & Environments of Health, University of Exeter, Exeter, UK; 5 Institute for Culture and Society, Western Sydney University, Sydney, Australia; 6 Department of Public Health, Environments & Society, London School of Hygiene & Tropical Medicine, London, UK; 7 Independent Research Consultant, Birmingham, UK

**Keywords:** Case study research, Context, Complex interventions, Public health, Health systems, Delphi approach, reporting principles

## Abstract

**Background:**

Guidance and reporting principles such as CONSORT (for randomised trials) and PRISMA (for systematic reviews) have greatly improved the reporting, discoverability, transparency and consistency of published research. We sought to develop similar guidance for case study evaluations undertaken to explore the influence of context on the processes and outcomes of complex interventions.

**Methods:**

A range of experts were recruited to an online Delphi panel, sampling for maximum diversity in disciplines (e.g. public health, health services research, organisational studies), settings (e.g. country), and sectors (e.g. academic, policy, third sector). To inform panel deliberations, we prepared background materials based on: [a] a systematic meta-narrative review of empirical and methodological literatures on case study, context and complex interventions; [b] the collective experience of a network of health systems and public health researchers; and [c] the established RAMESES II standards (which cover one kind of case study). We developed a list of topics and issues based on these sources and encouraged panel members to provide free text comments. Their feedback informed development of a set of items in the form of questions for potential inclusion in the reporting principles. We circulated these by email, asking panel members to rank each potential item twice (for relevance and validity) on a 7-point Likert scale. This sequence was repeated twice.

**Results:**

We recruited 51 panel members from 50 organisations across 12 countries, who brought experience of a range of case study research methods and applications. 26 completed all three Delphi rounds, reaching over 80% consensus on 16 items covering title, abstract, definitions of terms, philosophical assumptions, research question(s), rationale, how context and complexity relates to the intervention, ethical approval, empirical methods, findings, use of theory, generalisability and transferability, researcher perspective and influence, conclusions and recommendations, and funding and conflicts of interest.

**Conclusion:**

The ‘Triple C’ (Case study, Context, Complex interventions) reporting principles recognise that case studies are undertaken in different ways for different purposes and based on different philosophical assumptions. They are designed to be enabling rather than prescriptive, and to make case study evaluation reporting on context and complex health interventions more comprehensive, accessible and useable.

**Supplementary Information:**

The online version contains supplementary material available at 10.1186/s12874-023-01888-7.

## Background

Contemporary health system and public health challenges (e.g. tackling childhood obesity, improving access to mental health care for hard-to-reach groups) require complex delivery programmes that engage with issues of context. The interventions or programmes designed to tackle such challenges are complex with multiple, interconnected components delivered individually or targeted at communities or populations, and success is dependent on individuals’ responses and on the wider context. There is increasing realisation by evaluators, researchers, funders, policymakers and other users that meaningful evaluations of complex interventions need to tease out understanding of complex and dynamic relationships between context/s and intervention [1].

Case study research, involving in-depth exploration of phenomena in their natural, ‘real-life’ settings [2], can address these challenges. It is increasingly being used in evaluation of complex interventions [3]. This is because case study research enables the use of methods that support improved development and implementation in public health and health systems in ways that account for the contexts in which complex interventions are to be implemented.

### Why use case study research to evaluate complex interventions?

Many of the most pressing questions for public health research, where the focus is on system-level determinants [4, 5], and for health services research [6, 7], where provision and implementation varies across contexts [8], require methodological approaches that can account for complexity. Evidence about context and intervention is also crucial for questions of external validity. Policymakers, commissioners and other users require credible evidence of relevance to their contexts to perform ‘careful abstraction’ to the settings, populations [9], and ‘locales’ that matter for them [10].

Controlled (ideally, randomised) trials are widely accepted as the preferred design for maximising internal validity. However, experimental trial designs can have limited value in answering questions about the effects of interventions in complex systems [11, 12], or about transferability (how well the intervention works across different contexts) and generalisability (how well the intervention can be scaled up) [13, 14]. This recognition represents a fundamental shift, paving the way for research designs that are better placed to strengthen external validity and understanding of the relationship between intervention and context [15].

Empirical case studies typically enable a dynamic understanding of complexity (rather than restricting the focus on narrow problem delineations and simple fixes), and surface the different logics underpinning intervention implementation and effects [16, 17]. This is because they ‘*generally address multiple variables in numerous real-life contexts, often where there is no clear, single set of outcomes*’ ([8], p775). Case study research is therefore an important methodology for studying complexity and an invaluable resource for understanding the influence of real-world context on complex system-level interventions.

### Why are reporting principles needed?

Reporting guidelines such as CONSORT and PRISMA have improved the discoverability, transparency and completeness of reporting of RCTs [18] and systematic reviews [19] respectively. Moreover, there is increased emphasis on methodological pluralism in evaluation of complex interventions, combined with openness to broadening the evidence base to better understand both causality in and the transferability of system change interventions [6, 15, 20, 21]. Case study research evidence is essential to this, but can be difficult to find, is often under exploited and is variably reported [2].

Case study research is a diverse field, with multiple definitions and perspectives grounded in different ways of viewing the world, and involving different combinations of methods (see [2] for an overview). If evaluative health research is to progress debates on methods for understanding interventions as interruptions in complex systems [22], then we need to consider in more detail how researchers can conduct and report empirical case studies. This includes cutting across the methodological spectrum of case study research in ways that elucidate how the relationship between context and intervention might lead to particular effects. Recognising this, the UK Medical Research Council (MRC) Better Methods, Better Research panel funded the Triple C study, via a commissioned call, focused on developing guidance and reporting principles for *case study* research into the influence of *context* on *complex* health interventions. The Triple C study builds on existing frameworks and guidance, bringing together literature on conducting evaluations of complex interventions [23–28], on case study research [29] and on context [1].

Published literature, consultation with experts and our experience as trainers and mentors in case study research all suggest there is considerable diversity among evaluators, researchers, journal editors, peer reviewers and funders about what counts as high quality case study research and what it can tell us about context and complexity. The Triple C reporting principles and (forthcoming) guidance aim to advance the application of case study methodology as a means of evaluating complex system-level interventions and better understanding of the transferability of system change intervention.

## Methods

We developed reporting principles via an online Delphi panel. We followed an online adaptation of the Delphi method that we have used in previous studies to produce guidance on how to critically appraise research on illness narratives [30] and on standards for realist and meta-narrative evidence synthesis [31, 32]. Given the breadth of case study research, the Triple C study sought to surface conflict and agreement while mapping the spectrum of perspectives on case study research.

**Fig. 1:**
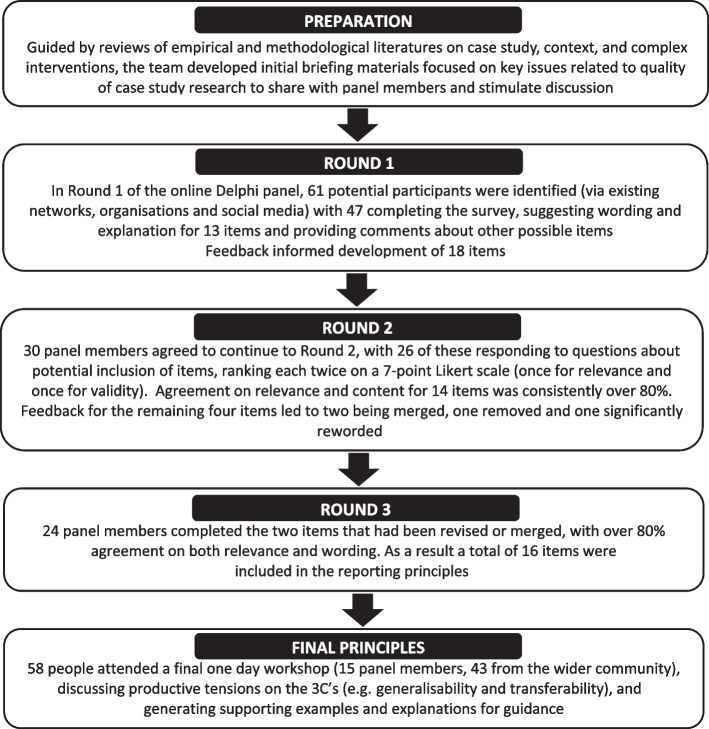
Overview of the Delphi process and development of Triple C reporting principles

The Delphi panel was conducted over three survey rounds (using an online survey tool), between September and December 2021 (see Fig. 1 for an overview). Ethics approval was obtained from the Medical Sciences Inter-Divisional Research Ethics Committee (IDREC) at the University of Oxford.

As is usual in Delphi studies, we sought to stimulate reflection and discussion amongst a panel of experts with a view to getting as close as possible to consensus and documenting the agreement reached as well as the extent of any residual disagreement [33]. We sought maximum variation of panel members to reflect diversity of disciplines, settings, sectors and experience. In line with the overall design of the phased study funded by the MRC, findings from the earlier systematic meta-narrative review of the methodological and empirical literature on case study research, context and complex interventions, and supporting reviews [2, 34], informed development of briefing materials for participants. These took the form of a list of 13 key issues related to quality of case study research in this field, including understanding and operationalising of context, to be considered when evaluating complex interventions: these were shared with panel members and additional issues sought (e.g. on the role of context in case study research).

In Round 1 we asked panel members to suggest wording for each item, indicate why they thought each item should/not be included, and suggest relevant theoretical and empirical papers. We used their feedback to construct a set of 16 items for Round 2 (including 4 from the established RAMESES II standards, that cover realist approaches [32]) in the form of questions, for potential inclusion in the reporting principles. We then asked panel members to rank each item twice on a 7-point Likert scale, once for relevance (i.e. should a question on this theme/topic be included at all in the guidance?) and once for validity (i.e. to what extent do you agree with this question as currently worded?) - see Supplementary file 1 for an example. Those who agreed that a question was relevant but disagreed on wording were invited to suggest changes to the wording. We also provided space for free text comments. In Round 3 we returned two items for further review.

### Recruitment and description of panel

We sought expressions of interest for panel members via the following routes: i) a network of over 50 international case study experts that we identified in the earlier stages of the study and contacted to inform scoping work (see [2] for detail), ii) representatives from funding bodies, journals and policy organisations (e.g. the MRC Better Methods Better Research Panel); and iii) via social media, making use of one author’s (TG) Twitter account to connect with a wider network of over 100,000 individuals, many involved in researching, evaluating, publishing and providing health care. This resulted in 65 potential participants who we then asked to provide further demographic information, along with setting, sector and experience of case study research. From this we recruited 61 panel members all of whom we invited to participate in the survey; 51 then clicked on the link for the survey, with 4 not progressing beyond the first page. Participants included academics, researchers, evaluators, policymakers and journal editors from over 50 organisations across 12 countries with diverse disciplinary backgrounds (covering anthropology, sociology, psychology, biomedical sciences, social geography, management and organisational studies, health economics, information systems, implementation science and a range of medical, dental and public health roles) and a range of experience of conducting, commissioning and publishing case study research in health-related settings. All either had been involved, or were planning, research using case study methodology to study complex health interventions.

We invited all panel members, along with a wider group, to a one-day workshop in February 2022 (held in Oxford, with an option to join remotely) where we sought additional feedback about the agreed principles. Fifty-eight people attended the event (excluding the study team), comprising 15 people who had participated in the Delphi panel and 43 drawn from the wider community of practice (including researchers, evaluators, funders, policymakers, editors and practitioners from within and outside of the UK and involved in developing, supporting, evaluating, funding and publishing work on complex interventions, including – but not limited to – case study research). Workshop participants actively contributed, through plenary sessions, panel debate and breakouts, to a discussion about the key findings from the study relating to context, case study and complexity. We used the Delphi panel as a starting point for identifying productive tensions to explore further in the workshop (e.g. relating to generalisability and transferability, operationalising context) and expand our understanding for the reporting principles, explanations and planned follow-on guidance. We did not change anything about the statements from what was scored in the Delphi, rather this process confirmed the reporting principles and provided additional explanation and examples on which to draw.

## Findings

### Overview of Delphi panel and collation of items

In Round 1 of the Delphi panel, 38 (of 47) members provided comments or suggestions for 13 items, with 32 members completing at least half of the survey. The first Round was time-intensive and many members faced time-pressures during the Covid-19 pandemic. We followed up with 30 members who had consented to continue to Round 2, who were presented with 13 items to rank. Panellists were also asked to comment on the inclusion of four additional items that we added, following analysis of free text comments in Round 1, and that were adapted from reporting principles for realist evaluation [32], relating to: Abstract; Ethical Approval and Considerations; Strengths and Limitations; and Funding and Conflict of Interest. In this second round 87% (*n*=26) of participants completed over half of the survey questions. Based on the rankings and free text comments our analysis indicated that two items needed to be merged, one item removed and one item significantly reworded. Minor revisions were made to the text of the other items based on the rankings and free text comments, and on the basis that agreement on relevance and content for these items was consistently over 80%. Following discussion among the study team we returned two items to Round 3 of the Delphi panel (Terminology, and Context, Complexity and relationship to the intervention), with 24 out of 26 panel members completing all items. Consensus was reached within three rounds, with over 80% agreement on both relevance and wording of 16 items (see Table 1). A summary of analysis across all three rounds of the Delphi can be found in Supplementary file 2.

**Table 1:** List of possible items to be included when reporting a case study evaluation of a complex intervention

Item			Reported in document Y/N/Unclear	Page(s) in documentation
TITLE				
1	Title	Is the term ‘case study’ used in the title and/or subtitle, index, key words, or abstract?		
ABSTRACT				
2	Abstract	In the abstract or summary, have the authors included brief details on: the policy, programme, intervention or initiative under evaluation; programme setting; purpose of the case study research; case study question(s) and/or objective(s); case study research strategy; data collection, documentation and analysis methods; key findings and conclusions?		
INTRODUCTION				
3	Terminology	Have the researchers described how they are using key terms related to case, context and complexity/complex intervention in their study, e.g. by including definitions, descriptions or examples? If no descriptions are provided, have the authors provided their reasons for not doing so?		
4	Philosophical bases			
5	Research questions	Have the authors set out clear research questions to be answered by their case study design?		
6	Rationale for doing case study research			
7	Context, complexity and relationship to the intervention	Have the authors: a) described how they have interpreted complexity, in relation to context, the intervention, and how they interact?; and b) explained how they have designed their study to investigate such complexity, including how complexity shaped the case?		
8	Ethical approval and considerations	Have the authors stated whether the case study research required and has gained ethical approval from the relevant authorities, and provided details as appropriate? If ethical approval was deemed unnecessary, have the authors explained why?		
9	Empirical methods	Have the authors described: a) how data were produced (when, by whom, from whom and how) and how they were analysed? b) how their methods relate to their research questions, design and approach? and c) how different data are integrated in the case analysis?		
RESULTS				
10	Findings	Have the authors presented their findings in ways that: a) convey sufficient richness to illuminate the case? b) provide justification for any interpretive inferences?		
11	Use of theory	Where authors have used theoretical concepts or frameworks in their case study research, have they described and justified these?		
DISCUSSION				
12	Generalisability and transferability	a) Have the authors explained any implications of their findings beyond their immediate context (e.g. in terms of their generalisability, transferability or usefulness)? b) If they stated that their findings have implications beyond their case, have the authors included sufficient information about the key contextual conditions and historical path-dependencies so that people can make informed judgements on the relevance of the findings for other contexts and settings?		
13	Researchers’ perspective and influence	Have the authors: a) offered critical reflections about how their position, status and perspectives may have shaped the research and the interpretation of findings? and b) included how the case study findings may have challenged their prior assumptions?		
14	Strengths and limitations	Have the authors discussed both the strengths of the case study design and its limitations? Have they included (but need not be limited to) considerations of all the steps in the case study evaluation processes?		
15	Conclusions and recommendations	Are the authors’ conclusions and recommendations supported by their findings? If relevant, have the authors considered the implications of their findings for current research, policy or practice?		
16	Funding and conflict of interest	Have the authors stated the funding source (if any) for the case study research, the role played by the funder (if any) and any conflicts of interests of the authors?		

Below we set out the Triple C reporting principles in more detail, informed by previous methodological publications [32] and guidance for developers of health research reporting guidelines [35]. After each item we include an explanation for its inclusion followed by an example drawn from published (publicly available) evaluations. The selection of examples was informed by our engagement with the case study literature in this field, discussion with workshop participants following the Delphi, and the review we conducted earlier in the study [2]. We have deliberately selected some of these examples enabling readers to link back to previous work describing the different disciplinary influences and methodological approaches allied to case study research. Examples are not intended as models for reporting relevant information about each item, but rather to illustrate how an item might be written up and to reflect different approaches to designing, developing, conducting, analysing and reporting case study research [2]. As text for examples has been extracted from publications, important contextual information will have been omitted and it may be necessary to consult the original publication to fully understand the evaluation it refers to. Details of additional materials suggested by panel members to support case study evaluations and the role of context in complex interventions are included in Supplementary file 3.

### TRIPLE C reporting principles

#### Item 1: Title

Is the term ‘case study’ used in the title and/or subtitle, index, key words, or abstract?

##### Example

“Improving quality and safety of care using "technovigilance": an ethnographic case study of secondary use of data from an electronic prescribing and decision support system” ([36] page 424).

##### Explanation

Our earlier review showed that some case study evaluations of complex interventions are not labelled as such in the title. Case study research involves a particular theoretical and methodological approach, and should be differentiated from other types of study (e.g. pragmatic trials, case reports). Adding the term “Case Study” or “Case study of a Complex Intervention” in the title of any publications may aid searching and identification. Knowledge users (e.g. researchers, policy makers) will be able to locate reports using case study research, and so build the interdisciplinary knowledge base for complex interventions. Where space or journal style does not permit use of such terms in the title, authors can use it in the index, keywords and/or abstract.

#### Item 2: Abstract

In the abstract or summary, have the authors included brief details on: the policy, programme, intervention or initiative under evaluation; programme setting(s); purpose of the case study research; case study question(s) and/or objective(s); case study research strategy; data collection, documentation and analysis methods; key findings and conclusions?

##### Example

“This article presents a case study of a project known as ‘Designing Better Health Care in the South’ that attempted to transform four separately incorporated health services in southern Adelaide into a single regional health service. The project's efforts are examined using Kotter's (1996) model of the preconditions for transformational change in organisations and the areas in which it met or failed to meet these preconditions are analysed, using results from an evaluation that was commenced during the course of the attempted reform. The article provides valuable insights into an attempted major change by four public sector health organisations and the facilitators and barriers to such change. It also examines the way in which forces beyond the control of individual public sector agencies can significantly impact on attempts to implement organisational change in response to an identified need. This case study offers a rare glimpse into the micro detail of health care reform processes that are so widespread in contemporary health services but which are rarely systematically evaluated” ([37] page 31).

##### Explanation

Authors will need to provide an abstract or summary (depending on the type of publication they are producing) that clearly describes the case study research. Apart from the title, a summary or abstract is often the only source of information accessible to searchers, and often used by literature reviewers to determine inclusion, so it is important to enable visibility and allow potential knowledge users to determine the relevance of the case study evaluation. The information contained in it needs to allow the reader to decide whether the evaluation is a case study evaluation of a complex intervention, the context in which it was conducted and relevance to their needs.

#### Item 3: Terminology

Have the researchers described how they are using key terms related to case, context and complexity/complex intervention in their study, e.g. by including definitions, descriptions or examples? If no descriptions are provided, have the authors provided their reasons for not doing so?

##### Example

“The content of the EQUIP intervention is based on an evolving conceptualization of equity-oriented care. Specifically, in previous research developed and conducted in partnership with PHC clinics and other organizations serving marginalized populations, we identified evidence- and theory-informed key dimensions of PHC services that position equity as an explicit goal… Through the prior empirical work, we developed a framework identifying (a) four key dimensions of equity-oriented PHC services, which are particularly relevant when working with marginalized populations, and (b) following from those key dimensions, 10 strategies to guide organizations in enhancing their capacity for equity-oriented services, as detailed elsewhere… Ongoing refinement of this framework led us to re-conceptualize inequity-responsive care as the overarching aim, and as foundational to supporting health and well-being through the provision of culturally safe care, trauma- and violence-informed care, and contextually tailored care (Fig. 1). Below, we briefly describe these key dimensions of equity-oriented services, which provide the basis for the EQUIP intervention components.” ([38] page 3) (See paper for detailed discussion of concepts).

##### Explanation

It is important that key terms are described and applied consistently throughout a publication, making it clear to readers how they have been used throughout the case study. Clarity and consistency will enable readers to interpret, compare and apply research findings appropriately. This is particularly important in relation to case definition, which is central to case study research and consequential for the knowledge produced. We know from our review that authors sometimes only offer a description of how a case was selected but not of how the case under study was defined, either in terms of the boundaries of the case or what the case represented (i.e., what is it a case of?) [2]. This is important as, for example, defining a case by mentioning ‘the health care institution’ at the exclusion of – for instance - policy, discourse and wider structural relations has consequences for what counts as evidence within the case and how the case might offer points of transferability to other contexts.

Given the diversity of case study research it is neither desirable nor possible to develop a shared vocabulary or set of definitions about case, context and complexity: different case studies will use different terms in different ways. Authors need to convey what they mean by the key terms relevant to their case study evaluation of a complex intervention. Depending on the approach adopted, authors may tend towards close definition or broader description of terms but, whatever terms are used, author(s) need to make clear and justify their approach in relation to the type of case study research and methodological approach they are using. Where this is not possible or desirable, authors need to provide an explanation as to why.

#### Item 4: Philosophical bases

Have the authors provided explanations about: a) what they assume about the nature of reality (ontology)? b) how they think they can find out about that reality (epistemology)? c) whether their methods follow from their assumptions?

##### Example

***“Theoretical Considerations***

A critical realist approach

The research was concerned with real services and real people. The intervention was based on a belief that the systems involved are complex and holistic – in the sense of involving layers of reality – and that these layers of interest are real if not always tangible. We wanted to understand the links between components of these layers of social reality and were less interested in a descriptive account of practitioners’ feelings and perceptions, which a postmodern approach might have sought.

Critical realism as espoused by Bhaskar (1998) and others has a universal theory of causation based on generative principles. It is the temporal conjunction of causative powers, which brings about the regularities seen in societies, or the transformation of such societies. The potential mechanisms of causation residing in both actors and society are real and present even when not active, and when actualized may or may not be observable (empirical). Whether or not an outcome or regularity occurs is determined by the interplay of positive and countervailing mechanisms. Critical realists recognize the importance of both individual agency and the influence of the structures and culture of society.

Critical realism therefore has a philosophical stance in keeping with the study of very real but complex and interacting phenomena involving individuals and society. It provided a basis to help describe how and why a complex social intervention did or didn’t work” ([39] page 72).

##### Explanation

Case studies make a particular contribution to the evidence base, driven by distinct ways of thinking about the nature of reality (ontology) and the nature of knowledge (epistemology). Such distinctions enable those reading case studies to assess the type and quality of the contribution being offered by the case study findings. Researchers therefore need to clearly express their assumptions embedded within the research design that have led them to make particular decisions and choices with regards to methodology and methods used, and the knowledge claims being made. There should be correspondence between the underlying philosophical approach and the methodological approach, methods and data analysis used in the case study research.

Ontological and epistemological approaches need to be reflected upon throughout case study research. This is especially relevant for case study research due to it being such a diverse, multi-disciplinary field in which multiple methods are available with varying philosophical roots ranging from positivism, critical realism, through to social realism and interpretivism (see our earlier review for an overview, especially Table 1 [2]).

Wherever possible authors need to make clear the ontological and epistemological assumptions underpinning their research in the publication. There are structural constraints at play that guide the level of detail about underpinning philosophical assumptions that is possible to provide in some publications (particularly those with limited word count or that do not typically publish case study research). Recognising these constraints, authors should report their case study, and the assumptions underpinning it, that is appropriate for different publications, purposes and audiences.

#### Item 5: Research questions

Have the authors set out clear research questions to be answered by their case study design?

##### Example

“The main objectives of this evaluation were the following: (1) to study the functioning of the project in relation to the actors, issues, and strategies used; (2) to better understand the results and effects of use on access, continuity and quality of services and work, service organization, and practice transformation; (3) to explore socio-political, regulatory, organizational, governance, clinical, professional, economic, legal and technological factors influencing implementation, adoption and use, and ultimately the sustainability and dissemination of telepathology; and (4) to identify conditions that may be useful to ensure better integration and diffusion of telehealth in health systems” ([40] page 423).

##### Explanation

Case study research examining the influence of context on complex interventions needs to reflect the underlying reason for choosing a case study design in the first place, and how it relates to the research questions that need answering. This will inevitably vary according to the focus of the study, type of case study research approach adopted and rationale (see ‘Item 6: Rationale for doing case study research’), and the ways in which both context and complexity are conceptualised (see ‘Item 3: Terminology’).

Case study evaluations of complex interventions cannot address all potential research questions or issues. The scope of the evaluation has to be clarified. This may involve discussion and negotiation with (for example) commissioners of the evaluation, context experts, research funders and/or users. The processes used to establish purpose(s), scope, questions, and/or objectives should be described.

Given the iterative nature of much case study research, if the questions, objectives and/or protocol changed over the course of the evaluation, it should be reported here or in ‘Item 10: Findings’.

#### Item 6: Rationale for doing case study research

Have the authors justified: (a) why they have chosen a case study design for their research? And (b) their particular approach, including in relation to literature on the methodology of case study research?

##### Example

For (b): “Our decision to use Stake (2005) rather than Yin (2009) as the methodologist to follow was based on our combined consideration of the intent of the research and our philosophical orientation. Yin presented a much more structured approach to case study research than did Stake. Some critics of his work have suggested that Yin’s research has been situated within a postpositivist paradigm, whereas Stake’s has been a constructivist” ([41] page 1268).

##### Explanation

Case studies of complex interventions vary in their approach and are influenced by a range of philosophical positions (e.g. positivism, critical realism, interpretivism). This means that while some case studies set out to evaluate complex interventions in real life settings, others interrogate how mechanisms are triggered in specific contexts and lead to particular outcomes, or adopt an emergent approach and use theory-building to surface complexity. For instance, realist evaluation is rooted in a social realist philosophy and places particular emphasis on understanding generative causation (in this case, understanding how complex interventions generate outcomes) and how causal mechanisms are shaped and constrained in different contexts. This makes it particularly suitable for evaluations of certain topics and questions – for example, complex social programmes that involve human decisions and actions. Naturalistic case studies are rooted in an interpretivist philosophy of science, and use ‘thick description’ [42] of (typically) a small number of cases to understand the non-linear unfolding of events and actions and the ways in which these shape, and are shaped by, changing contexts. This, combined with a dynamic appreciation of the relationships between context and complex intervention, make it particularly suitable for evaluations of multi-faceted social programmes, involving multiple actors in dynamic relationships that shift over time.

The intent of this item is that the relevance of the case study research approach to the evaluation research question should be made explicit. The authors’ rationale for using case study research should clarify what they mean by case study, and explain the appropriateness of using case study for an evaluation of a complex intervention.

Published case studies demonstrate that some researchers have deliberately adapted or been ‘inspired’ by the case study research approaches set out by the methodologists Robert K. Yin and Robert Stake. The description and rationale for any adaptations made to these or any other approach and how they have shaped the evaluation of a complex intervention should be provided. Where evaluation approaches have been combined, authors should articulate their approach (in relation to case study literature, and wider literature where relevant), with the implications for methods made explicit. Such information will allow assessment and debate amongst researchers, users, commissioners and editors on the suitability of those adaptations for the purposes of evaluating a specific complex intervention and recognising the influences of context.

#### Item 7: Context, complexity and relationship to the intervention

Have the authors: a) described how they have interpreted complexity, in relation to context, the intervention, and how they interact?; and b) explained how they have designed their study to investigate such complexity, including how complexity shaped the case?

##### Example

“At the core of complexity theory is the notion that individual properties differ from collective properties […] The theory holds that this difference results from the interactions that occur between and among parts of a collective, between and among parts and collectives, and between and among collectives. The theoretical picture this creates is one of overlapping systems that have some coherence but that are also linked to, and part of, other systems that are continually adapting to each other. This focus on the interrelationships of a complex system as being central to causal processes, gives reason to think that complexity theory may hold some useful ways to think about complex social processes” ([43] page 223).

##### Explanation

What the authors mean by ‘context’, and its theorised relationship to complexity and intervention, needs to be specified when reporting case studies of complex interventions. This makes it possible to understand how different kinds of contexts are conceptualised in the same study, how they compare (e.g. the ‘context’ of a specific hospital versus the policy ‘context’) or the relationship between context and intervention. These concepts are hard to define in a useful way, hence the emphasis in this item is on describing and explaining them instead. An authors’ description of context and complexity will inevitably be informed by the theories, approaches or frameworks that they use in their research. For instance, case studies analysing change in organisations might focus on structural and cultural factors that shape the characteristics of a service; while those using realist evaluation will consider the relationship of context/s to mechanism and outcome. Naturalistic case studies might focus on history and path dependency, evaluate how people interact with an intervention, and assess how structure and context continuously shape and re-shape the intervention.

There are many ways to conceive of and operationalise context, which has implications for how evaluations of complex interventions are designed and conducted, the knowledge produced and potential transferability. An influential definition from the MRC guidance refers to context as ‘*anything external to the intervention which impedes or strengthens its effects*’ ([10] p2). This intervention-centred approach reflects concerns (e.g. of researchers, funders) to prepare the grounds for an intervention, plan implementation and assess transferability across settings. Another approach sees context as relational and dynamic, and as emerging over time in multiple different levels of the wider system - context is seen as the numerous opportunities, constraints, issues and happenings that become salient as the intervention unfolds. From this perspective, context cannot be conceptualised and ‘measured’ separately from the intervention.

#### Item 8: Ethical approval and considerations

Have the authors stated whether the case study research required and has gained ethical approval from the relevant authorities, and provided details as appropriate? If ethical approval was deemed unnecessary, have the authors explained why?

##### Example

“This study is part of a larger project “A realist evaluation of the antiretroviral treatment adherence club programme in selected primary health-care facilities in the metropolitan area of Western Cape Province, South Africa”, which has received ethics clearance from the University of the Western Cape Research Ethics Committee (UWC REC) (Registration No: 15/6/28). In addition, we obtained ethical clearance from the Provincial Department of Health of the Western Cape Province. Furthermore, we obtained the permission of the facility head and management before data collection processes commenced.

At the level of the study participants, we first provided the interviewed participants with an information sheet of the project. This was followed by a verbal explanation of the role of the participant and the significance of their participation. They were required to sign an informed consent form. We promised and ensured confidentiality and anonymity by identifying the participants using pseudo names and by password-protecting all files related to the study.” ([44] page 8)

##### Explanation

Case study research of complex interventions is a form of primary research that usually involves human participants. The research must be conducted ethically. Case study researchers come from a range of different professional backgrounds and disciplines, and work in diverse fields. This means that different professional ethical standards and both national and local ethics regulatory requirements may well apply. Researchers should ensure that they are aware of and comply with their professional obligations and ethics requirements throughout their case study research.

Some case study research (especially, but not only, naturalistic case studies) is emergent in terms of the design and conduct of the research. This means that legitimate changes may be required to the methods used and participants recruited as the evaluation of a complex intervention, and relationship between context/s and intervention, evolves. Anticipating that such changes may be needed is important when seeking ethical approval. It is helpful to build in flexibility to case study evaluations of complex interventions to allow for updating ethics approvals and for explanation of emergence and adaptation to those who provide ethics approvals.

#### Item 9: Empirical methods

Have the authors described: a) how data were produced (when, by whom, from whom and how) and how they were analysed? b) how their methods relate to their research questions, design and approach? and c) how different data are integrated in the case analysis?

##### Example

“[Data collection] resulted in a sizeable data-set comprising interviews collected at four time points over a seven-year period. All interviews were transcribed in full. They were analysed using an approach informed by the constant-comparative method, but with specific attention directed towards certain issues identified a priori and included in the topic guide. These were informed by the literature, by our knowledge of the case-study sites, and by two theoretical frameworks that provided ‘sensitizing concepts’ around the challenges of sustaining organizational change.23,24 Themes were thus developed both inductively and deductively, to cover issues derived from the literature, our prior work and existing conceptual frameworks, but also issues that emerged from close, repeated readings of the data sources; data were coded to these themes by SW, and were then analysed by GPM and SW, first on a case-by-case basis, and then across themes. The other authors then each analysed selected themes according to their own expertise, ensuring the validity of the initial coding and interpretation, and adding their own insights that further developed the analysis.” ([45] page 192)

##### Explanation

Research users need to understand where case study data have come from and how they have been analysed to be able to interpret the findings about the relationship between context and complex interventions. In order for evaluations to be transparent authors should clearly specify the empirical methods they used in their case study.

Case study designs often draw on a range of methods, data sources and analytic techniques. Outputs from case study research are commonly developed through methods such as synthesis or triangulation. Together the range of methods and approaches that make up case study methodology need to be clearly described. The process of recruiting sites and participants to an evaluation of a complex intervention, and how the sample of selected participants contributed to the development of the case needs to be explained to readers. Data collected and/or generated, and how these were analysed, need to be carefully described in relation to the research questions adopted as well as the case, context and complexity. Clarity about how specific datasets contributed to analysis is just as important as explaining how data were collected. This includes clearly articulating how ontological and epistemological assumptions about the case were operationalised within the analysis to produce the findings being reported. The exact approach will be guided by the case study design and philosophical foundations (suggested methodological texts are provided in Supplementary file 3, and are provided in addition to the material cited in the main paper).

#### Item 10: Findings

Have the authors presented their findings in ways that: a) convey sufficient richness to illuminate the case? b) provide justification for any interpretive inferences?

##### Example

“Results are presented on the basis of the study’s three principal questions, starting with the municipal context, followed by local public-health policies, and finally their relation to the SNPHP. The identified local health policies included alcohol- and drug-prevention, long-term sick-leave rehabilitation, and anti-bullying measures. Two local public-health policies were given more explicit attention, and are here described in greater detail: the policy process for the new overall goals for all municipal activities in Municipality A, and the revised community-wide alcohol program in Municipality B” ([46] page 222).

##### Explanation

Findings can be presented in a range of different ways, again guided by the case study design and philosophical foundations. For instance, case studies that develop and test interventions might present findings in a structured format, and include a diagrammatic model of links between intervention and outcome. Case studies that analyse change in organisations often present findings in a narrative format allowing authors to surface the dynamic organisational, policy or human backdrop to organisational change and how this changes over time as the intervention is implemented. Whichever approach is adopted, this should align with the overarching case study research design and include sufficient detail for readers to assess the coherence, plausibility and relevance of the case study findings.

In their presentation of findings authors need to clearly reflect their understanding of the context of the study, the case study methodology and the study aim(s). The context for the evaluation of the complex intervention will likely already have been described (if not, it should be described here), and authors should now focus on how context has shaped the findings of the case study, and the complex intervention, including temporal influences. Findings of the case study evaluation, and how they relate to the wider class of phenomena under investigation, should be clearly explained. Any supporting information should be clearly included, with sufficient data (or worked analysis) to substantiate and illuminate within or cross case analysis. Where relevant, disagreements or challenges faced by the researchers in making any inferences should be reported here.

#### Item 11: Use of theory

Where authors have used theoretical concepts or frameworks in their case study research, have they described and justified these?

##### Example

“Our paper illuminates the impact of social prescribing on health inequalities by exploring the classed contexts shaping clients' experiences of a social prescribing intervention in the North of England. We use Bourdieu's concepts of habitus, field and capital as a lens through which to analyse how practices of client engagement are connected to class. We pay particular attention to the spatio-temporal nature of everyday practice to explore how class enables and constrains participation in social prescribing interventions” ([47] No page number available).

##### Explanation

Case study evaluations of complex interventions aim to build on what is already known about a particular programme or initiative, adding to a cumulative body of knowledge about that intervention (or class of interventions) and with findings assessed in relation to the theoretical perspectives from which they derive and to which they may contribute. Theories arrange sets of concepts to help us define and explain, in this case, the influence of context on complex interventions [36]. Use of theory enables researchers to move beyond basic description to in-depth analysis, interpretation and explanation.

All research engages with theory in some way. It might be explicitly aiming to develop theory, it might be using particular theoretical frameworks to structure the research or it may be using theoretical concepts as sensitising devices to guide data collection and analysis. In all cases one of the objectives of using theory is to enable transferability and generalisability beyond the case. How theory has been used (or the reasons why authors do not wish to explicitly use any theory) needs to be set out.

The use of theory to guide research design, data collection, analysis and reporting is crucial to building a robust evidence base regarding complex interventions. Authors need to be explicit about their theoretical choices. This will enable researchers, commissioners, funders and users to build on their work and generalise findings. The extent of discussions about theory, and the location of that discussion, will depend on the use and purpose of theory for the case study evaluation, the type of publication (and space typically available for theoretical development and discussion) and the case study approach adopted. For instance, some authors may focus on the role of theory in developing and testing hypotheses, others in developing programme theory or as a means of developing dialogue with other studies and literature. If theoretical propositions have been developed, then authors should state both the initial theoretical propositions and the final propositions.

If theoretical development is a goal of the study, authors should describe how findings elucidate or test the authors’ chosen theory.

#### Item 12: Generalisability and transferability

a) Have the authors explained any implications of their findings beyond their immediate context (e.g. in terms of their generalisability, transferability or usefulness)? b) If they stated that their findings have implications beyond their case, have the authors included sufficient information about the key contextual conditions and historical path-dependencies so that people can make informed judgements on the relevance of the findings for other contexts and settings?

##### Example

“This article has discussed some effects identified from an evaluation of the natural experiment of free bus travel for young people in London, UK. We have summarized evidence […] that it enhanced social inclusion without reducing the amount of active travel; and that it made a contribution to ‘destigmatizing’ bus transport, an important precondition of reducing private car use. We argue that these effects are likely to hold in other settings where there is an efficient and accessible bus service (i.e. one that is perceived as offering a density of routes and frequent reliable services) and where the scheme is a universal, rather than conditional, entitlement” ([48] page 401).

##### Explanation

Our previous review showed a historical tendency to understate and critique the potential of case study research to offer explanation and to test or build theory, compounded by the historical relegation of case study research to the bottom of a methodological hierarchy of effectiveness [49]. While acknowledging that not all case studies of complex interventions aim for generalisability, authors should make clear how findings can be generalised theoretically or applied to other settings in some way. How this is done will depend on case study design, and can range from: aggregating and standardising datasets resulting from multiple data collection activities into lists of 'contextual factors' to explain variation in intervention outcomes; to acknowledging that generalisability of findings is limited by the extent to which contexts are similar; to using concepts such as ‘demi-regularity’ to convey the idea of partial transferability. In some instances, case study findings are seen as informing programme theories—that is, theories that are sufficiently detailed to help explain some regularities in empirical findings but which do not account for every eventuality. Those conducting single case studies emphasise naturalistic generalisability of a richly-described ‘n of 1’ case and the development and refinement of substantive theory (with a clear sense that theory is needed to justify generalisability). Whichever approach is adopted, authors need to differentiate the generalisability or transferability of multiple, synthesised quantitative and qualitative findings in case studies of complex interventions.

#### Item 13: Researchers’ perspectives and influences

Have the authors: a) offered critical reflections about how their position, status and perspectives may have shaped the research and the interpretation of findings? and b) included how the case study findings may have challenged their prior assumptions?

##### Example

“Our work resonates with a number of trends in American community psychology. Most importantly we locate ourselves firmly within the tradition of community-based action research (Israel, Eng, Schultz, & Parker, 2005; Minkler & Wallerstein, 2003). All our work is conducted in partnership with research communities, with the explicit aims of working collaboratively with local people to identify possibilities for action towards improved health and well-being, and strategies for implementing such action.

We also locate our work within the context of on-going debates about how best to create social settings that enable health. Broadly speaking, the goals of our work are very much in the spirit of Kelly’s ecological approach to community psychology (Kelly, 2006; Kelly, Ryan, Altman, & Stelzner, 2000, chap. 7) with its emphasis on the importance of developing settings that support individuals in building both personal and social resources to address pressing life challenges. …

Conforming to linear protocol of an academic paper, we provide an account of our conceptual framework at the beginning of this paper. However, we must emphasise that this framework represents the evolving conceptualisation that has emerged over the course of the community engagement we outline below. Whilst this framework has its roots in our earlier work on HIV/AIDS in other South African contexts cited above (Campbell, 2003; Campbell et al., 2004, 2005a, b), it has been considerably honed and fine-tuned through our practical experience in Entabeni.” ([50] page 350) (See paper for references).

##### Explanation

A researcher’s perspective can shape the conduct and reporting of case studies. It is therefore critical to explicitly describe any relationships between individual researchers, the case and its context. Authors should describe and, wherever possible, critically reflect on their own position and perspective in relation to the case and how this may have contributed to the research, including how the empirical findings may have challenged tacit or prior assumptions. Depending on the approach adopted, the nature of the complex intervention and context being studied, this might involve describing researchers’ backgrounds in approaching the study, emphasizing their prior understandings of the phenomena under study (e.g. interviewers, analysts or research team), and position within the study (e.g. researcher; researcher and implementer). As a suggestion, prior understandings relevant to the analysis could include, but are not limited to, descriptions of researchers’ demographic and/or cultural characteristics, credentials, experience with phenomena, training, values and how these shape decisions about the design, conduct, analysis and interpretation of case study materials.

Reflections need not be included as a separate item but could be offered in different parts of a publication (e.g. when discussing context, strengths and limitations), depending on type of publication and institutional constraints. They paint a picture for the reader about the case study research process with as much information as possible about how it was conducted and by whom, and can also provide reassurance to readers about any potential conflicts of interest (see ‘Item 16: Funding and conflict of interest’).

#### Item 14: Strengths and limitations

Have the authors discussed both the strengths of the case study design and its limitations? Have they included (but need not be limited to) considerations of all the steps in the case study evaluation processes?

##### Example

***“Limitations and strengths***

In Longsight, the separation of priority setting and strategic issues in the CFG, and working on joint action on the resulting agenda through the CWG, appeared important in enabling effective working. However, whilst we thought we had explained this aspect of the CE model to stakeholders and participants on numerous occasions, there appeared limited understanding of its intended operation when discussed in evaluation interviews. The division of strategic and operational aspects between the CFG and CWG was often unclear to those who were not routinely dealing with strategic issues. It seems that stakeholders needed to trust that this did work, without necessarily having an interest in why or how it worked. In retrospect, detailing the CE model and collecting all relevant materials together on a project internet site may have provided a more accessible source of information than printed, distributed materials” ([51] page 2876).

##### Explanation

The strengths and limitations of case study methodology, its application and utility in evaluating complex interventions and the influence of context should be discussed. There are inherent limits to any methodological approach in the real world due to, for example, constraints on time and resources, the skill mix and collective experience of the researchers, and/or by anticipated or unanticipated challenges in gathering the data or the data itself. Depending on the approach adopted, case study researchers evaluating complex interventions may face particular challenges collecting certain types of information (e.g. about mechanisms, which cannot usually be directly observed), or evidencing findings (e.g. about the relationships between context, mechanism and outcome). General limitations should be made explicit, along with specific limitations relevant to the particular case study approach and ways of conceptualising and operationalising complexity and context used, so that readers can interpret the findings in light of them. Strengths (e.g. being able to build on emergent findings by iterating the evaluation design) or limitations imposed by any modifications made to the case study research design and processes, the complex intervention or the context for the case study should also be reported and described.

Discussion about strengths and limitations is typically included in the discussion section of publications, but may need to be provided earlier in some evaluation and/or journal reporting styles (e.g. as part of methods).

#### Item 15: Conclusions and recommendations

Are the authors’ conclusions and recommendations supported by their findings? If relevant, have the authors considered the implications of their findings for current research, policy or practice?

##### Example

“Automation offers a variety of tangible benefits and is often proposed as a means to increase patient safety. But, as this case demonstrates, automation also creates new vulnerabilities, some with substantial consequences. Emergent vulnerabilities, such as arise from the interaction among disparate, independently designed components, seem almost impossible to foresee in anything other than the most general terms. Health care seems especially vulnerable to these sorts of threats for several reasons: (1) the relative youth of complex computer application in the field; (2) the general unfamiliarity of health professionals and managers with methods for reducing vulnerabilities; (3) the fragmented nature of health care “organizations”; (4) the potential subversion of risk information into internal, conflicting agendas; and (5) the lack of formal or regulatory frameworks promoting the assessment of many types of new technologies. These factors are as much social-organizational as they are technological. As we consider increased automation in health care, we should pay as much attention to anticipating new vulnerabilities and the social component of the sociotechnical system, and to introducing well-established design and engineering risk assessment methods into the field as we do to the anticipated benefits [12]” ([52] page 1500). (See paper for references).

##### Explanation

A clear line of reasoning is needed to link the conclusions drawn from case study findings as presented in the results section of any publication. Some authors may prefer to present their conclusions alongside their data (i.e. in ‘Item 10: Findings’). Authors should be clear about how their conclusions were drawn from the case study findings and on how they think relevant contextual elements shape these conclusions.

Any recommendations made need to reflect and emerge from the purpose of the study, its context and the findings, and consider what conclusions mean for policy and practice. For example, if the evaluation of a complex intervention is small-scale or preliminary, or if the strength of evidence is limited, firm implications for practice and policy may be inappropriate.

Case study evaluations of the kinds discussed here are intended to inform the design, development, implementation, adoption and routinisation of an intervention. In many evaluations there will be an expectation to provide guidance on future directions for one or more of these. The particular implications arising from the influence of the context on the intervention should be reflected in these discussions.

If recommendations are given, these should be consistent with the case study research approach adopted. Recommendations should especially take account of context as understood in the particular study. For example, if an evaluation found that an intervention worked for some people or in some contexts, it would be inappropriate to recommend that it be run everywhere for everyone in the same way.

All conclusions and recommendations should be supported by clear evidence and any nuances that would affect the value and relevance of any recommendations when transferred to other contexts need to be clearly set out with supporting evidence.

#### Item 16: Funding and conflict of interest

Have the authors stated the funding source (if any) for the case study research, the role played by the funder (if any) and any conflicts of interests of the authors?

##### Example

**“Competing interests**

The authors have no financial competing interests to declare. Two co-authors (MU and OGS) participated in the improvement team at the hospital.…

**Acknowledgments**

The Swedish Vinnvård Research programme funded the work of Pamela Mazzocato and Johan Thor. The authors thank Krister Eriksson, Åsa af Jochnick, and Sara Saltin for their help with collection of quantitative data, Lena Nord and Anna Ekman for inviting us to study the improvement work and for giving access to the data, and all patients and staff who contributed to this study at the Danderyd Hospital” ([53] page 7).

##### Explanation

The source of funding for an evaluation of a complex intervention and/or personal conflicts of interests may influence the case study questions, methods, data collection and analysis, conclusions and/or recommendations. No evaluation is a ‘view from nowhere’, and readers will be better able to interpret the evaluation if they know why it was done, how it was funded and for which commissioner.

If an evaluation is published, the process for reporting funding and conflicts of interest as set out by the publisher should be followed.

## Discussion

In this paper we have presented findings from a Delphi study in the form of reporting principles for case study evaluation of the role of context in complex interventions. Our aim is to support clarity of reporting of the diversity of case study research. We acknowledge the wide variation in case study research and its implementation. The history of case study methodology and the array of disciplines involved have produced a rich and varied literature, and case study evaluations of complex health interventions also differ significantly in their epistemological, theoretical and methodological foundations [2, 3]. We sought to develop principles that can be useful for researchers, commissioners, practitioners and publishers, building on an earlier meta-narrative review of the literature on case study, context and complex interventions, and supporting reviews. This allowed us to embrace the multiplicity of approaches and applications of case study research in this field.

There is currently limited information in health systems and public health research about the diversity of available case study research approaches and how these can (variably) support implementation and evaluation of complex interventions in health care. There are also a number of papers that utilise the term ‘case study’ as a description (e.g. in the title or abstract), for qualitative or mixed-methods studies addressing context and complex interventions but which are not designed as case study research and do not engage with case study methodology. This is legitimate in that the paper may present a case study of a particular phenomenon, but it does not necessarily report ‘case study research’. It raises questions about classification and reporting of case studies and case study research, which we hope that the reporting principles will be helpful in addressing. It is unclear if and how these guidelines will limit the (legitimate) use of ‘case study’ in titles of works that are not aiming to use ‘case study methodology’.

The Delphi technique was an ideal research tool for the study. Although the Delphi approach aims at ‘consensus’, the objective is not to force a ‘definitive answer’ but rather to develop ‘possible solutions’ ([54], p353) and to explore the extent of both consensus and conflict amongst experts on a given issue. Consulting with experts was a necessary step in developing meaningful guidance and reporting standards and in challenging and extending our own – varied and situated - views as an interdisciplinary research team via engagement with a broader community of scholars in the field. We sought Delphi participants from a range of disciplinary, experiential and geographic settings – while we managed significant breadth, this was skewed towards those from North America, Europe and Australia. As we were unable to specify which panel members completed the final Delphi rounds (a requirement of ethics approval which precluded Internet Protocol (IP) tracking for the online survey), this may have introduced some bias into the Delphi process. While we reached a high level of consensus via the Delphi process, and subsequent workshop, there was also some residual disagreement. We reported this non-consensus and the nature of the dissent in the explanations included in the findings section. In doing so our aim is, not only to make such dissent explicit, but also to expose inherent (philosophical or practical) ambiguities in case study research and acknowledge that not everything can be resolved.

### How to use the Triple C reporting principles

Reporting principles are intended to aid evaluators, researchers, editors, commissioners, policy makers and other users to know and understand what needs to be reported when writing up case study evaluations of complex interventions. The list of items provided in Table 1 is intended as an overview to help guide that reporting, with the final two columns (informed by the PRISMA 2020 statement [55] and earlier RAMESES II reporting standards [32]) included as a means of indicating where in a document each item has been reported.

The Delphi panel were generous in their feedback and comments. A recurrent theme was the call to frame items as reporting *principles*, not standards. This was due to the diversity of case study research approaches and variety of methods used, and concerns by some (but not all) panel members that narrow interpretation of items might lead to an emphasis on certain kinds of case study research (e.g. positivist approaches to ‘test’ complex interventions) over others, and/or to limits on publication diversity (with health science journals generally perceived to be less interested in case study research than other types of research designs). With this in mind, the items listed in Table 1 should be interpreted flexibly depending on the purpose of the evaluation, place of publication and needs of the audience/users. The steer from panel members, which we strongly support, is that not all reports of case study evaluations of complex interventions need necessarily be reported in the same way. Our intention in providing these principles is therefore to offer directions for improving the discoverability, transparency, consistency and quality of case study reporting, rather than to provide a prescriptive checklist of items that need to be included in any report of case study research. The findings from our earlier systematic meta-narrative review of the literature further support this, pointing to at least four main approaches to case study research [2], each grounded in different epistemological positions that guide approach, methods and, crucially, reporting.

The reporting principles set out what might be expected for each item. However, authors (and editors) will need to exercise judgement about how much information is needed. The information reported should be sufficient to enable readers to judge that a case study evaluation has been planned, executed, analysed and reported in a coherent, trustworthy and plausible fashion, both against the guidance set out within an item and for the overall purposes of the evaluation itself. The reporting principles are not intended to provide detailed guidance on the conduct of case study research (as part of the Triple C project we are also developing detailed guidance, which will be available as a separate publication).

In the Delphi panel and subsequent workshop discussion, several participants commented that the publication of guidance in this area may have the unintended effect of promoting rationalistic and technocratic approaches to reporting at the expense of the diversity of case study research and situational judgements by study teams about context and complexity. For this reason, the items set out in Table 1 should be seen as a starting point for reflection and discussion, not a substitute for it, when editors, reviewers and knowledge users consider the issues arising when case study evaluations of complex interventions are planned, designed, conducted and disseminated. Our intention, with this paper and other outputs from the study, is to provide a resource that users (evaluators, funders and so on) can easily access and use to support the design, conduct and reporting of case study research. We will disseminate these widely via formal channels (e.g. publication, panel discussion, advisory committees) and informal debate (e.g. via social media). While the principles are intended for those involved in evaluating complex health interventions, we recognise that case study research draws on diverse disciplines, most notably anthropology and sociology, and that they may therefore have wider relevance and applicability.

## Conclusion

The above reporting principles have evolved from the MRC-funded Triple C study, focused on Case study, Context and Complex interventions. A previously published meta-narrative review, plus scoping reviews, informed the Delphi process reported here and the development of reporting principles that recognise that case studies are undertaken in different ways for different purposes and based on different philosophical assumptions.

The Triple C reporting principles list 16 items to be considered when reporting a case study evaluation of a complex intervention. These are designed to be enabling rather than prescriptive, and to make reporting of case study evaluations of complex interventions more comprehensive, accessible and useable.

## Supplementary Information

**Additional file 1. **Example question from Round 2 of the Delphi process.

**Additional file 2. **Results from Triple C Delphi Panel, Rounds 1-3.

**Additional file 3. **Suggested materials put forward by panel members to support case study evaluations and the role of context in complex interventions.

**Additional file 4. **Qualitative comments during the Delphi process.

## Data Availability

The datasets generated and/or analysed during the current study are not publicly available due to consent being given by participants for the purposes of this study only. De-identified datasets are available from the corresponding author on reasonable request.
