# Supplementary material for: TRIPLE C reporting principles for case study evaluations of the role of context in complex interventions
Source: BMC Med Res Methodol. 2023 May 13;23:115. doi: 10.1186/s12874-023-01888-7 (PMC10182844; doi:10.1186/s12874-023-01888-7)
Supplement: Supplementary file 1 — Additional file 1. Example question from Round 2 of the Delphi process. [file 12874_2023_1888_MOESM1_ESM.docx]

**Supplementary file 1: Example question from Round 2 of the Delphi process**
