# Supplementary material for: TRIPLE C reporting principles for case study evaluations of the role of context in complex interventions
Source: BMC Med Res Methodol. 2023 May 13;23:115. doi: 10.1186/s12874-023-01888-7 (PMC10182844; doi:10.1186/s12874-023-01888-7)
Supplement: Supplementary file 2 — Additional file 2. Results from Triple C Delphi Panel, Rounds 1-3. [file 12874_2023_1888_MOESM2_ESM.docx]

| **Supplementary file 2: Results from Triple C Delphi Panel, Rounds 1-3** | | | |
| --- | --- | --- | --- |
| **TRIPLE C DELPHI PANEL ROUND ONE:**  **Case Study Research into the Role of Context in Complex Health Interventions** | | | |
| TOPICS | RESPONSES | | |
|  | **YES**  % (n) | **NOT SURE**  % (n) | **NO**  % (n) |
| **1. TERMINOLOGY** | | | |
| **Include** (N=38) | **81.6% (31)** | 18.4% (7) | - |
| **2. PHILOSOPHICAL BASES OF CASE STUDY RESEARCH ON COMPLEX INTERVENTIONS** | | | |
| **Include** (N=35) | **60.0 (21)** | 34.3 (12) | 5.7 (2) |
| **3. CLASSIFICATION** | | | |
| **Include** (N=35) | **65.7 (23)** | 25.7 (9) | 8.6 (3) |
| **4. TITLE** | | | |
| **Include** (N=34) | **58.8 (20)** | 20.6 (7) | 20.6 (7) |
| **5. RATIONALE FOR USING CASE STUDY RESEARCH** | | | |
| **Include** (N=33) | **90.9 (30)** | 9.1 (3) | - |
| **6. USE OF THEORY** | | | |
| **Include** (N=32) | **62.5 (20)** | 34.4 (11) | 3.1 (1) |
| **7. CONTEXT, COMPLEXITY AND RELATIONSHIP TO THE INTERVENTION** | | | |
| **Include** (N=33) | **75.8 (25)** | 18.2 (6) | 6.1 (2) |
| **8. CASE STUDY METHODOLOGY** | | | |
| **Include** (N=33) | **48.5 (16)** | 39.4 (13) | 12.1 (4) |
| **9. DATA COLLECTION AND ANALYSIS METHODS IN CASE STUDIES** | | | |
| **Include** (N=31) | **90.3 (28)** | 9.7 (3) | - |
| **10. FINDINGS** | | | |
| **Include** (N=31) | **80.7 (25)** | 12.9 (4) | 6.5 (2) |
| **11. GENERALISABILITY, TRANSFERABILITY AND SPREAD** | | | |
| **Include** (N=31) | **74.2 (23)** | 19.4 (6) | 6.5 (2) |
| **12. ACKNOWLEDGING RESEARCHERS’ PERSPECTIVE AND INFLUENCE** | | | |
| **Include** (N=30) | **73.3 (22)** | 20.0 (6) | 6.7 (2) |
| **13. CONCLUSIONS AND RECOMMENDATIONS** | | | |
| **Include** (N=31) | **74.2 (23)** | 22.6 (7) | 3.2 (1) |

| **TRIPLE C DELPHI PANEL ROUND TWO:**  **Case Study Research into the Role of Context in Complex Health Interventions** (N=26, except where indicated) | | | | | | | | |
| --- | --- | --- | --- | --- | --- | --- | --- | --- |
|  | **RATINGS** | | | | | | |  |
| TOPICS | **(1)**  **Strongly**  **Disagree**  % (n) | **(2)**  **Mostly disagree**  % (n) | **(3)**  **Slightly**  **Disagree**  % (n) | **(4)**  **Not**  **Sure**  % (n) | **(5)**  **Slightly**  **Agree**  % (n) | **(6)**  **Mostly**  **Agree**  % (n) | **(7)**  **Strongly Agree**  % (n) | **Median**  **(IQ Range)** |
| **1. TITLE** | | | | | | | | |
| **Relevance** | **-** | - | - | 3.8 (1) | 7.7 (2) | 30.8 (8) | **57.7 (15)** | 7.0 (1.0) |
| **Content** | - | 3.8 (1) | 3.8 (1) | 3.8 (1) | 3.8 (1) | 30.8 (8) | **53.8 (14)** | 7.0 (1.0) |
| **2. TERMINOLOGY** | | | | | | | | |
| **Relevance** | **-** | 3.8 (1) | - | - | - | 23.1 (6) | **73.1 (19)** | 7.0 (1.0) |
| **Content** | **-** | 3.8 (1) | 7.7 (2) | 11.5 (3) | 3.8 (1) | 26.9 (7) | **46.2 (12)** | 6.0 (2.25) |
| **3.** **PHILOSOPHICAL BASES** | | | | | | | | |
| **Relevance** | **-** | 7.7 (2) | - | 11.5 (3) | 11.5 (3) | **34.6 (9)** | **34.6 (9)** | 6.0 (2.0) |
| **Content** | **-** | 11.5 (3) | - | 7.7 (2) | 3.8 (1) | **42.3 (11)** | 34.6 (9) | 6.0 (1.25) |
| **4. RESEARCH QUESTIONS** | | | | | | | | |
| **Relevance** | **-** | - | - | - | 7.7 (2) | 11.5 (3) | **80.8 (21)** | 7.0 (0) |
| **Content** (N=25) | **-** | - | 4.0 (1) | 4.0 (1) | 4.0 (1) | 20.0 (5) | **68.0 (17)** | 7.0 (1.0) |
| **5. RATIONALE FOR DOING CASE STUDY RESEARCH** | | | | | | | | |
| **Relevance** | - | - | - | - | 15.4 (4) | 19.2 (5) | **65.4 (17)** | 7.0 (1.0) |
| **Content** | - | - | 7.7 (2) | 7.7 (2) | 3.8 (1) | 23.1 (6) | **57.7 (15)** | 7.0 (1.0) |
| **6. CONTEXT, COMPLEXITY AND RELATIONSHIP TO THE INTERVENTION** | | | | | | | | |
| **Relevance** | - | - | 3.8 (1) | 7.7 (2) | 15.4 (4) | 23.1 (6) | **50.0 (13)** | 6.5 (2.0) |
| **Content** | 7.2 (2) | - | 19.2 (5) | 11.5 (3) | 11.5 (3) | 19.2 (5) | **30.8 (8)** | 5.5 (4.0) |
| **7. EMPIRCAL METHODS** | | | | | | | | |
| **Relevance** | **-** | - | 3.8 (1) | - | - | 3.8 (1) | **92.3 (24)** | 7.0 (0) |
| **Content** | **-** | 3.8 (1) | 3.8 (1) | 3.8 (1) | 11.5 (3) | 30.8 (8) | **46.2 (12)** | 6.0 (1.25) |
| **8. FINDINGS** | | | | | | | | |
| **Relevance** | **-** | - | 3.8 (1) | 7.7 (2) | 3.8 (1) | 23.1 (6) | **61.5 (16)** | 7.0 (1.0) |
| **Content** | **-** | - | 15.4 (4) | 7.7 (2) | 15.4 (4) | **30.8 (8)** | **30.8 (8)** | 6.0 (2.25) |
| **9. USE OF THEORY** | | | | | | | | |
| **Relevance** | **-** | - | 3.8 (1) | 3.8 (1) | 3.8 (1) | 7.7 (2) | **80.8 (21)** | 7.0 (0) |
| **Content** (N=25) | **-** | 4.0 (1) | 4.0 (1) | 4.0 (1) | - | 20.0 (5) | **68.0 (17)** | 7.0 (1.0) |

| TOPICS | **(1)**  **Strongly**  **Disagree**  % (n) | **(2)**  **Mostly disagree**  % (n) | **(3)**  **Slightly**  **Disagree**  % (n) | **(4)**  **Not**  **Sure**  % (n) | **(5)**  **Slightly**  **Agree**  % (n) | **(6)**  **Mostly**  **Agree**  % (n) | **(7)**  **Strongly Agree**  % (n) | **Median**  **(IQ Range)** |
| --- | --- | --- | --- | --- | --- | --- | --- | --- |
| **10. GENERALISABILITY AND TRANSFERABILITY** | | | | | | | | |
| **Relevance** | - | - | - | - | 7.7 (2) | 19.2 (5) | **73.1 (19)** | 7.0 (1.0) |
| **Content** | - | 3.8 (1) | 3.8 (1) | - | 19.2 (5) | 26.9 (7) | **46.2 (12)** | 6.0 (2.0) |
| **11. TIME AND SPREAD** | | | | | | | | |
| **Relevance** (N=25) | **-** | 8.0 (2) | 4.0 (1) | - | 12.0 (3) | **40.0 (10)** | 36.0 (9) | 6.0 (1.5) |
| **Content (**N=25) | **-** | 12.0 (3) | 24.0 (6) | 8.0 (2) | 8.0 (2) | 24.0 (6) | 24.0 (6) | 5.0 (3.5) |
| **12. ACKNOWLEDGING RESEARCHERS’ PERSPECTIVE AND INFLUENCE** | | | | | | | | |
| **Relevance** (N=25) | **-** | - | 4.0 (1) | - | 16.0 (4) | **40.0 (10)** | **40.00 (10)** | 6.0 (1.0) |
| **Content** (N=25) | **-** | 4.0 (1) | - | - | 20.0 (5) | **44.0 (11)** | 32.0 (8) | 6.0 (1.5) |
| **13. CONCLUSIONS AND RECOMMENDATIONS** | | | | | | | | |
| **Relevance** | **-** | 3.8 (1) | - | - | - | 23.1 (6) | **73.1 (19)** | 7.0 (1.0) |
| **Content** | 3.8 (1) | - | - | 7.7 (2) | 3.8 (1) | 30.8 (8) | **53.8 (14)** | 7.0 (1.0) |

| **TRIPLE C DELPHI PANEL ROUND THREE:**  **Case Study Research into the Role of Context in Complex Health Interventions** | | | | | | | | |
| --- | --- | --- | --- | --- | --- | --- | --- | --- |
|  | **RATINGS** | | | | | | |  |
| TOPICS | **(1)**  **Strongly**  **Disagree**  % (n) | **(2)**  **Mostly disagree**  % (n) | **(3)**  **Slightly**  **Disagree**  % (n) | **(4)**  **Not**  **Sure**  % (n) | **(5)**  **Slightly**  **Agree**  % (n) | **(6)**  **Mostly**  **Agree**  % (n) | **(7)**  **Strongly Agree**  % (n) | **Median**  **(IQ Range)** |
| **2. TERMINOLOGY (Revised)** | | | | | | | | |
| **Content** (N=25) | 4.0 (1) | - | 8.0 (2) | - | - | 36.0 (9) | **52.0 (13)** | 7.0 (1.0) |
| **6. CONTEXT, COMPLEXITY AND RELATIONSHIP TO THE INTERVENTION (Revised)** | | | | | | | | |
| **Content** (N=24) | 8.3 (2) | - | - | 4.2 (1) | 8.3 (2) | **41.7 (10)** | 37.5 (9) | 6.0 (1.0) |
