# Supplementary material for: TRIPLE C reporting principles for case study evaluations of the role of context in complex interventions
Source: BMC Med Res Methodol. 2023 May 13;23:115. doi: 10.1186/s12874-023-01888-7 (PMC10182844; doi:10.1186/s12874-023-01888-7)
Supplement: Supplementary file 3 — Additional file 3. Suggested materials put forward by panel members to support case study evaluations and the role of context in complex interventions. [file 12874_2023_1888_MOESM3_ESM.docx]

## Supplementary file 2: Suggested materials put forward by panel members to support case study evaluations and the role of context in complex interventions

1. Abma TA, Stake RE. (2014) Science of the Particular:An Advocacy of Naturalistic Case Study in Health Research. Qualitative Health Research; 24(8):1150-61.
2. Alpi, K.M. and Evans, J.J., 2019. Distinguishing case study as a research method from case reports as a publication type. *Journal of the Medical Library Association*: JMLA, 107(1), p.1.
3. Anthony, S. and Jack, S. (2009) Qualitative case study methodology in nursing research: an integrative review. *Journal of Advanced Nursing* 65(6): 1171-1181.
4. Anderson, R. A., et al. (2005) Case study research: the view from complexity science. *Qualitative Health Research* 15(5): 669-685.
5. Appleton, J. V. (2002). "Critiquing approaches to case study design for a constructivist inquiry." *Qualitative Research Journal* 2(2): 80-97.
6. Atkins, C., & Sampson, J. (2002). Critical appraisal guidelines for single case study research. *European Conference on Information Systems*, 1987; 100–109.
7. Ayres, L., et al. (2003) Within-case and across-case approaches to qualitative data analysis. *Qualitative Health Research* 13(6): 871-883.
8. Baškarada, S. (2014) Qualitative Case Study Guidelines. *Qualitative Report*, 19(40), 1–25.
9. Baxter, P., & Jack, S. (2008). Qualitative Case Study Methodology: Study Design and Implementation for Novice Researchers. *Qualitative Report*, 13(4), 544-559.
10. Bergen, A. and A. While (2000) A case for case studies: exploring the use of case study design in community nursing research. *Journal of Advanced Nursing* 31(4): 926-934
11. Bozeman, B. and H. K. Klein (1999) The case study as a research heuristic: lessons from the R&D value mapping project. *Evaluation and Program Planning* 22: 91-103.
12. Burgess, R. G., et al. (1994) Four studies from one or one study from four. Multi-site case study research. Analysing qualitative data. A. Bryman and R. G. Burgess. London, Routledge.
13. Burawoy M. The Extended Case Method. Sociological Theory. 1998;16(1, March 1998.
14. Byrne, D. (2013) Case-Based Methods: Why We Need Them; What They Are; How To Do Them. In Byrne, D., & Ragin, C. C. (eds.). The Sage handbook of case-based methods (pp. 1-10). Sage Publications.
15. Çakmak, Z. and Akgün, İH (2018) A Theoretical Perspective on the Case Study Method. *Journal of Education and Learning*, 7(1), pp.96-102.
16. Carolan CM, Forbat L, Smith A. (2016) Developing the DESCARTE model: the design of case study research in health care. *Qualitative Health Research*; 26(5):626–39.
17. Clark AM. (2013) Theorizing approaches to parts, powers and the whole intervention. Social Science & Medicine; 93:185-93.
18. Cope, D. G. (2015) Case study research methodology in nursing research. *Oncology Nursing Forum*, Oncology Nursing Society.
19. Cowley, S., et al. (2000) Generalising to theory: the use of a multiple case study design to investigate needs assessment and quality of care in community nursing. *International Journal of Nursing Studies* 37: 219-228.
20. Crowe S, Cresswell K, Robertson A, Huby G, Avery A, Sheikh A. (2011) The case study approach. BMC Medical Research Methodology; 11:100.
21. Cunningham, JB. (1997) Case study principles for different types of cases. *Quality and Quantity* 31: 401-423.
22. Curtis, S., et al. (2000) Approaches to sampling and case selection in qualitative research: examples in the geography of health. *Social Science and Medicine* 50: 1001-1014.
23. Damianakis, T., & Woodford, M. (2012) Qualitative research with small connected communities: Generating new knowledge while upholding research ethics. *Qualitative Health Research*, 22, 708–718.
24. Darke, P., et al. (1998) Successfully completing case study research: combining rigour, relevance and pragmatism. *Information Systems Journal* 8: 273-289.
25. Dopson, S. (2003) The potential of the case study method for organisational analysis. *Policy and Politics* 31(2): 217-226.
26. Dyer, W. G. and A. L. Wilkins (1991) Better stories, not better constructs, to generate better theory: a rejoinder to Eisenhardt. *Academy of Management Review* 16(3): 613-619.
27. Eisenhardt, K. M. (1989) Building theories from case study research. *Academy of Management Review* 14(4): 532-550.
28. Eisenhardt, K. M. (1991) Better stories and better constructs: the case for rigor and comparative logic. *Academy of Management Review* 16(3): 620-627.
29. Ellis, L. B. (2003) Illuminative case study design: a new approach to the evaluation of continuing professional education. *Nurse Researcher* 10(3): 48-59.
30. Flyvbjerg, B. (2006). Five Misunderstandings About Case-Study Research. Qualitative Inquiry, 12(2), 219–245.
31. Fàbregues S, Fetters MD. )2019) Fundamentals of case study research in family medicine and community health. Fam Med Community Health. 2019;7(2):e000074..
32. Gangeness, J. and E. Yurkovich (2006) Revisiting case study as a nursing research design. *Nurse Researcher* 13(4): 7-18.
33. Gerring (2004) What is a case study and what is it good for? *American Political Science Review*; 98(2): 341-354
34. Gerring (2007) Case Study Research: Principles and Practices. Cambridge: Cambridge University Press.
35. Gilgun, JF. (1994) A case for case studies in social work research. *Social Work* 39(4): 371-380.
36. Gomm, R., Hammersley, M. and Foster, P. (2000) Case study and generalisation. In: Gomm, R.; Hammersley, M. and Foster, P. (eds) *Case Study Method: Key Issues, Key Texts.* London: Sage, pp. 98–115.
37. Gomm, R., et al. (2000). Case study method. Key issues, Key Texts. London, Sage publications.
38. Grant, A., Bugge, C. & Wells, M. (2020) Designing process evaluations using case study to explore the context of complex interventions evaluated in trials. *Trials* 21, 982.
39. Gray, M. (1998) Introducing single case study research design: an overview. *Nurse Researcher* 5(4): 15-24.
40. Hamel, J., et al. (1993) Case study methods. Newbury Park, Sage.
41. Harrison, H., Birks, M., Franklin, R., & Mills, J. (2017) Case Study Research: Foundations and Methodological Orientations. Forum Qualitative Sozialforschung / Forum: Qualitative Social Research, 18(1).
42. Hatcher, W., McDonald, B. D., & Brainard, L. A. (2018). How to write a case study for public affairs. *Journal of Public Affairs Education*, 24(2), 274–285.
43. Huws, U., & Dahlmann, S. (2007) Quality standards for case studies in the European Foundation, Dublin: European Foundation for the Improvement of Living and Working Conditions.
44. Hyett, N., et al. (2014) Methodology or method? A critical review of qualitative case study reports." International Journal of Qualitative Studies on Health and Well-being 9(1): 23606.
45. Ingleton, C., et al. (1997) Multidisciplinary case study as an approach to the evaluation of palliative care services: two examples. *International Journal of Palliative Nursing* 3(6): 335-339.
46. Iwakabe, S., & Gazzola, N. (2009) From single-case studies to practice-based knowledge: Aggregating and synthesizing case studies. *Psychotherapy Research*, 19(4–5), 601–611.
47. Jensen, J. L. and R. Rodgers (2001) Cumulating the intellectual gold of case study research." *Public Administration Review* 61(2): 235-246.
48. Greenhalgh, J. & Manzano, A. (2021) Understanding ‘context’ in realist evaluation and synthesis, International Journal of Social Research Methodology, DOI: [10.1080/13645579.2021.1918484](https://doi.org/10.1080/13645579.2021.1918484)
49. Keen, J. and T. Packwood (1995) Qualitative research: case study evaluation. *British Medical Journal* 311: 444-446.
50. Kohn, L. T. (1997). Methods in case study analysis. The Centre for Health System Change.
51. Lincoln, Y. S. and E. G. Guba (2002) Judging the quality of case study reports. In Huberman and Miles. *The Qualitative Researchers Companion*. Thousand Oaks, Sage.
52. Luck, L., et al. (2006) Case study: a bridge across the paradigms. *Nursing Inquiry* 13(2): 103-109.
53. Macpherson, I. et al. (2000) Case study in the contemporary world of research: using notions of purpose, place, process and product to develop some principles for practice. *International Journal of Social Research Methodology* 3(1): 49-61.
54. McGloin, S. (2008) The trustworthiness of case study methodology. *Nurse Researcher* 16(1).
55. Meier, N. & Dopson, S. (2019) Context in Action and How to Study It - Illustrations from Health Care. Oxford: Oxford University Press.
56. Merriam, S. B. (1998). Qualitative research and case study applications in education. San Francisco, Josey-Bass publishers.
57. Meyer, C. B. (2001) A case in case study methodology. *Field methods* 13(4): 329-352.
58. Miles, R. (2015) Complexity, representation and practice: Case study as method and methodology. *Issues in Educational Research* 25(3): 309-318.
59. Mitchell JC (1984). Case studies. In: Ellen RF, editor. Ethnographic Research: A Guide to General Conduct. London: Academic Press.
60. Noor, K. B. M. (2008) Case study: A strategic research methodology. *American journal of applied sciences* 5(11): 1602-1604.
61. Ormrod, S. (2003) Organisational culture in health service policy and research: "third way" political fad or policy development?" *Policy and Politics* 31(2): 227-237.
62. Paparini etc Petticrew, M., Greenhalgh, T., Hanckel, B. and Shaw, S., 2020. Case study research for better evaluations of complex interventions: rationale and challenges. BMC medicine, 18(1), pp.1-6.
63. Paparini, S., Green, J., Papoutsi, C. *Murdoch, J. Petticrew, M. Greenhalgh, T. Hanckel, B. & Shaw SE.* Case study research for better evaluations of complex interventions: rationale and challenges. *BMC Medicine* 18, 301.
64. Paparini, S., Papoutsi, C. Murdoch, J. Green, J., Petticrew, M. Greenhalgh, T. & Shaw SE. (2021) Evaluating complex interventions in context: systematic, meta-narrative review of case study approaches. BMC Medical Research Methodology 21, 225.
65. Pawson R, Tilley N. (1994) Realistic evaluation. London / Thousand Oaks, California: Sage
66. Pfadenhauer, L.M., Gerhardus, A., Mozygemba, K. et al. (2017) Making sense of complexity in context and implementation: the Context and Implementation of Complex Interventions (CICI) framework. *Implementation Science* 12, 21.
67. Ragin CC & Becker HS (Eds)(1992) What is a case?: exploring the foundations of social inquiry. Cambridge : Cambridge University Press.
68. Riege, AM (2003) Validity and reliability tests in case study research: a literature review with "hands-on" applications for each research phase. *Qualitative Market Research* 6(2): 75-86.
69. Rosenberg JP & Yates PM (2007). Schematic representation of case study research designs." *Journal of Advanced Nursing* 60(4): 447-452.
70. Ruffa, C. (2020) Case Study Methods: Case Selection and Case Analysis, ch 6 in Curini and Franzese, The SAGE Handbook of Research Methods in Political Science and International Relations. London, Sage Publications.
71. Sandelowski, M. (1996) One is the liveliest number: the case orientation of qualitative research. *Research in Nursing and Health* 19(6): 525-529.
72. Sandelowski, M. (2011) ‘Casing’’ the Research Case Study. *Research in Nursing & Health*, 34(2), pp. 153–159.
73. Segar J, Checkland K, Coleman A, McDermott I. (2015) Thinking about Case Studies in 3-D: Researching the NHS Clinical Commissioning Landscape in England. Case Study Evaluation: Past, Present and Future Challenges. Advances in Program Evaluation. 15: Emerald Group Publishing Limited; p. 85-105.
74. Sharp, K. (1998) The case for case studies in nursing research: the problem of generalisation. *Journal of Advanced Nursing* 27: 785-789.
75. Sharp JL, et al. (2012) A mixed methods sampling methodology for a multisite case study. *Journal of mixed methods research* 6(1): 34-54.
76. Stake RE. (1995) The art of case study research. London: Sage Publications Ltd.
77. Stake, R. E. (2000). Case studies. In Denzin & Lincoln, Handbook of qualitative research. Thousand Oaks, CA, Sage Publications, pps 435-454.
78. Stake, RE. (2000). The case study method in social inquiry. Case study method. Key issues, key texts. R. Gomm, M. Hammersley and P. Foster. London, Sage.
79. Stake, R. E. (2005). Qualitative Case Studies. In N. K. Denzin & Y. S. Lincoln (Eds.), The Sage handbook of qualitative research, 3rd edition, (pp. 443–466). Thousand Oaks, CA: Sage Publications Ltd.
80. US General Accounting Office (1990). Case Study Evaluations. Washington DC.
81. Walshe, C. (2011) The evaluation of complex interventions in palliative care: An exploration of the potential of case study research strategies. *Palliative Medicine*, 25(8), pp.774-781.
82. Wells, M., Williams, B., Treweek, S. et al. Intervention description is not enough: evidence from an in-depth multiple case study on the untold role and impact of context in randomised controlled trials of seven complex interventions. *Trials* 13, 95 (2012).
83. Yazan, B. (2015). Three Approaches to Case Study Methods in Education: Yin, Merriam, and Stake. *Qualitative Report*, 20(2), 134-152.
84. Yin RK (2014) Case Study Research Design and Methods . Thousand Oaks, CA: Sage.
85. Yin RK. Enhancing the quality of case studies in health services research. Health services research. 1999;34(5 Pt 2):1209.
