# Supplementary material for: TRIPLE C reporting principles for case study evaluations of the role of context in complex interventions
Source: BMC Med Res Methodol. 2023 May 13;23:115. doi: 10.1186/s12874-023-01888-7 (PMC10182844; doi:10.1186/s12874-023-01888-7)
Supplement: Supplementary file 4 — Additional file 4. Qualitative comments during the Delphi process. [file 12874_2023_1888_MOESM4_ESM.docx]

## Supplementary file 3: Qualitative comments during the Delphi process

##### Title, abstract and terminology

“*It is good practice to include the phrase "Case Study" or "Case study of a Complex Intervention" in your title if you would like your research to be retrieved in online databases and subsequently used and cited*.”

“*I am not sure what you mean, besides 'please don't call your work case study research if it is not case study research' and 'calling your work case study research means you need to argue for this design, what it is, what it can/can't do, what it is a case study of, how the case/s were selected and why, and what all these choices allows you to do/research'*”

“*So as to aid the accessibility of your project to researchers and clinicians, we request that authors consider including a clear indication of methodology and setting*…”

“*Clearly defining the application of key terms (case, context, complexity) is important for case study reporting standards; we expect authors to include these definitions in their work.*”

“*A point of a researcher defining terms is to consider what the language is making room for. It's useful to have a shared vocabulary, but not necessarily vital. The presumption that we share one leaves us worse of*.”

##### Philosophical basis, and rational for using case study research

“*It is important that readers know where you are coming from as researchers, in terms of what you believe about the nature of reality (ontology) and the nature of knowledge (epistemology). Case studies make a particular contribution to the evidence base, which needs to be clearly understood by those reading it. Researchers therefore need to clearly express their assumptions embedded within the research design which have led them to make particular decisions and choices with regards to methodology and methods used*.”

“*Most researchers have an underlying set of assumptions about how they view the world and knowledge. This can influence how research is planed, undertaken and analysed*.”

“*The researchers perspective on the nature of the world, knowledge and contextualization can help those who consume case studies to interpret the findings*.”

“*The rationale for using case study approaches for the author's particular research question is an important part of detailing case study methodology and should be included as such, as well as reflected in the author's positionally (philosophical basis).*”

“*Prospective authors need to have examples of how to evaluate a methodologies appropriateness, and its limitations*.”

“[Its important to include] *the rationale for using case study methodology instead of other qualitative methodologies*.”

##### Research questions, ethical approval and empirical methods

“*The diversity of the case study setting and research questions necessitates a methodology that captures the uniqueness of each scenario. The different methodological approaches need to be fully understood to enable careful justification and selection of the most suitable methodology*.”

“*It is imperative that you clearly describe your methodology. If you do not do this, the reader will be unclear about what you did and why, and may not be able to trust the findings you present*.”

“[Its important to] *describe site, participants, time frame, unit of observation, unit of analysis, and analytical approach* [and] *give details of ethical considerations and stakeholder involvement in the study*.”

“*We often pay close attention to how data were identified and collected, but it is often the case that less attention is paid to how data contributes to analysis or, to be simple, what role the data you collect answer the questions you pose. Being clear about how the data you collect contributes to your analysis is just as important as explaining how your data were collected*.”

“*In multiple case study research has the approach to cross-case analysis been justified and described*?”

##### Context, complexity and relationship to the intervention

“*The context, the intervention, the complexity of the context and the complexity of the intervention need to be clearly understood and described, as well as the relationship between them. This rich understanding of the overall scenario underpins the methodological detail, and reporting and interpretation of study findings*.”

“*I am not sure about Relationship to the Intervention - research should be Intervention-centric, and context and complexity be related to them*.”

“*There are concentric circle ways of thinking on it, the giant checklist people, the events-in-systems people. Honestly you cannot dictate but you can invite people to be chose what fits for them*.”

“*Interventions occur in systems and systems sit within contexts. Interventions, systems and context may each hold characteristics of complexity. The simplicity/complexity of each needs to be articulated as well as their relationship to each other*.”

“*This might be addressed as part of the 'terminology' as well as linked to the theoretical perspective being taken by the researchers*.”

##### Findings, and use of theory

“*The use of theory to guide research design, data collection, analysis and reporting is crucial to building a robust evidence base regarding complex interventions. Being explicit about your theoretical choices will enable others to build on your work and generalise your findings*.”

“*Theory may underpin the methodological approach, or contribute to the development or enhancement of theory*.”

“*The theory underpinning a case study is valuable to understanding the application of case study methodology and to the interpretation and degree of generalization of the results*.”

“*The great strength of an evaluation of a complex intervention is that what could be learned from it can be useful to others, and what is useful to others is something that can be replicated in similar settings--and that is the value of infusing theory into evaluations*.”

“*Findings can be presented in a range of different ways. It is important to ensure there is enough detail for readers to assess their coherence, plausibility and relevance***.”**

“*A findings section should be just that--a section that depicts findings. But the question becomes, "What do I include in my findings?" The answer is to re-state the purpose of the research and to state clearly to the reader what you are looking for, why, and how the facts of the case will be used to inform an analysis*.”

##### Generalisability and transferability

“*It is important to consider the 'logical' inferences that can be made from your research and applied elsewhere*.”

“*For quantitative results, it is indicated whether statistical generalizability is given. In the case of qualitative results, it is stated whether there is analytical generalizability or transferability in the sense of a case-to-case translation*.”

“*Societal progress is dependent on the build-up of credible scientific knowledge, and all researchers should be contributing to that incremental knowledge creation. Only by being clear and explicit in defining your Context can your findings be considered for relevance and transfer elsewhere*.”

“*It is important for researchers to be explicit about how they anticipate their research to be used*.”

“*Somewhat flippantly: Why should other people take your findings seriously*?”

##### Researchers’ perspective and influence

“*So many factors will influence the assumptions made, and observations recorded by a researcher during a case study. We need to acknowledge the researchers' perspective and influence to understand this*.”

“*Complexity in case study research is relational to the researcher and their stance*.”

“*I think this should focus on perspective and related bias*.”

“*It is important for readers to be able to draw conclusions about research with as much information as possible about how it was conducted and by whom. Describing yourself in relation to your research topic can help provide reassurance to readers that you do not have any conflicts of interest*.”

##### Strengths and limitations, conclusions and recommendations

**“***The implications of undertaking a case study is the evidence synthesised for influencing policy and practice. The conclusions need to reflect the study findings and are shaped by the context of the study***.”**

“*Not sure I would be too prescriptive here. I feel that the questions so far have not addressed temporality; that when you do case study research, it is in a specific moment in time and in a specific context, and it is really important to acknowledge this - not only as a limitation, but as a feature of the findings*.”

“*What do your findings mean for your local context? How has it changed practice, or will it*?”
